# IL-1β, IL-6, TNF- α and CRP in Elderly Patients with Depression or Alzheimer’s disease: Systematic Review and Meta-Analysis

**DOI:** 10.1038/s41598-018-30487-6

**Published:** 2018-08-13

**Authors:** Ada Ng, Wilson W. Tam, Melvyn W. Zhang, Cyrus S. Ho, Syeda F. Husain, Roger S. McIntyre, Roger C. Ho

**Affiliations:** 1 Department of Internal Medicine, National University Health System, Singapore, Singapore; 2 Alice Centre for Nursing Studies, Yong Loo Lin School of Medicine, National University of Singapore, Singapore, Singapore; 3 Biomedical Institute for Global Health Research and Technology, National University of Singapore, Singapore, Singapore; 4 Department of Psychological Medicine, National University Health System, Singapore, Singapore; 5 Institute of Medical Science, University of Toronto, Toronto, ON Canada; 6 Mood Disorders Psychopharmacology Unit, University Health Network, Toronto, ON Canada; 7 Department of Psychiatry, University of Toronto, Toronto, ON Canada; 8 Department of Toxicology and Pharmacology, University of Toronto, Toronto, ON Canada

## Abstract

We carried out systematic review and meta-analysis to evaluate whether peripheral levels of pro-inflammatory markers including Interleukin-1 beta (IL-1β), Interleukin-6 (IL-6), Tumor Necrosis Factor-α (TNF- α) and C-Reactive Protein (CRP) are significantly higher in elderly with depression and Alzheimer’s disease. We searched Pubmed, PsycINFO and Embase, and thirty-four relevant studies (2609 with Depression, 1645 with Alzheimer’s disease and 14363 Controls) were included. Compared with controls, IL-1β (pooled standardized mean difference [SMD]: 0.642; 95% confidence interval [CI]: 0.078–1.206; significant heterogeneity: I^2^ = 86.28%) and IL-6 (pooled SMD: 0.377; 95% CI: 0.156–0.598; significant heterogeneity: I^2^ = 88.75%) were significantly elevated in depression. There was no difference in TNF-α (p = 0.351) and CRP (p = 0.05) between those with depression and controls. Compared with controls, IL-1β (pooled SMD: 1.37, 95% CI: 0.06–2.68, significant heterogeneity: I^2^ = 96.01%) was significantly elevated in Alzheimer’s disease. There were no differences in IL-6 (p = 0.138), TNF-α (p = 0.451) and CRP (p = 0.07) between elderly with Alzheimer’s disease and controls. After Bonferroni adjustment, only IL-6 remained significantly higher in depression. Elderly with depression have higher IL-6 than controls, while those with Alzheimer’s disease did not have higher peripheral inflammatory markers.

## Introduction

Depression and Alzheimer’s disease are the most common psychiatric disorders amongst people aged 60 years and above. In recent years, depression resulted in 5.7% of Years Lived with Disability (YLD) in people above 60 years old and the annual global cost of dementia was US$604 billion^1^. In the future, the health burden due to depression and dementia will continue to increase because the number of people aged 60 years and above is expected to increase from 901 million in 2015 to 1.4 billion in 2030^2^. Furthermore, elderly with depression and dementia are associated with higher risk of suicide^3^, chronic obstructive lung disease^4^, stroke^5,6^ and pneumonia^7^, resulting in premature death. Dementia does not only affect elderly themselves but also affects the mental health of their caregivers^8^, leading to loss of productivity in the society^9^. It is thus prudent to understand the pathophysiology behind depression as well as Alzheimer’s disease which is one of the most common subtypes of dementia in elderly.

Inflammation has long been thought to play a vital role in the pathophysiology of psychiatric disorders in general adults^10^. In depression, the inflammatory response system (IRS) activates the hypothalamic-pituitary-adrenal (HPA) axis, leading to production of corticotropin-releasing hormone (CRH) and adrenocorticotropic hormone (ACTH), as well as increase in turnover of serotonin and catecholamines^11^. The IRS is driven by pro-inflammatory cytokines that are produced by macrophages, T cells and Natural Killer cells in response to immune activation^12^. The psycho-neuro-inflammatory theory was supported by previous studies which showed that stimulation of the HPA-axis resulted in the release of CRH by pro-inflammatory cytokines such as interleukin (IL)-1^13^, IL-6 and tumor necrosis factor (TNF-α)^14^. Since then, multiple studies demonstrated increased levels of acute phase proteins and cytokines such as C-reactive protein (CRP), IL-1β and IL6 in depression^15–20^. Elevation in peripheral inflammatory cytokines were also seen in other psychiatric disorders such as: IL-6, TNF-α and TGF-β in acute psychosis;^21^ IL-2R^22^ and IL-6^23,24^ in schizophrenia; IL-1β^25,26^, IL-6 and TNF-α^26^ in panic disorders; IL-1β and TNF-α in obsessive compulsive disorders^27^, and IL-1β, IL-6 and TNF-α in post-traumatic stress disorder (PTSD)^28,29^. The elevation of peripheral IL-1β, IL-6 and TNF-α could be a potential biomarker of vulnerability for psychiatric disorders in adults but their roles remain unknown for elderly.

Inflammation may play a pathological role in psychiatric disorders in elderly because a pro-inflammatory state is associated with ageing. Aging facilitates the pro-inflammatory state by disrupting peripheral immune system leading to excessive innate immune activity with release of pro-inflammatory cytokines and decrease in anti-inflammatory molecules^30^. The disruption in the periphery-central nervous system (CNS) immune communication via the blood-brain barrier (BBB) partly attributed by the peripheral injury further contributes to the pro-inflammatory shift of CNS. There is also altered discordant CNS response to inflammation with the upregulation of genes controlling inflammatory processes, as demonstrated by an increase in pro-inflammatory gene expression in late life of mice^31^ and humans^32^. Microglia and astrocytes are the two main cell components of the CNS that play a pivotal role in the central neuro-inflammatory process responsible for neuro-biological hemostasis. With the pro-inflammatory shift of the aging CNS, there is proliferation of both activated microglia and astrocytes resulting in continuous production of pro-inflammatory cytokines^33^. This process is further amplified in depression and Alzheimer’s disease, whereby chronic stress^34^ and amyloid beta (Aβ)^35^ respectively disrupts the microglia activity, leading to further release of the pro-inflammatory cytokines, interleukins and chemokines. In addition, depression and Alzheimer’s disease cause alteration in the BBB, allowing permeability of the peripheral inflammatory cells into the CNS^36^, further enhancing a state of neuro-inflammation with consequent tissue damage. For depression, Martinez-Cengotitabengoa *et al*.^37^ performed a systematic review and found that elevation of peripheral IL-8, IL-6, and TNF-α could indicate vulnerability to late-life depression. This review was based on qualitative review of 6 studies and the findings require further verification by a meta-analysis. For all-cause dementia, a meta-analysis found that elevation of peripheral IL-6 and CRP levels were associated with increased risk of developing dementia^38^. Nevertheless, another meta-analysis found that there was no significant difference in mean CRP levels between elderly with and without Alzheimer’s disease^39^. The role of TNF-α as causative and or as contributor to pathogenesis of dementia remains inconclusive^40,41^.

Hitherto, no meta-analysis has specifically compared the peripheral levels of proinflammatory markers including IL-1β, IL-6, TNF-α and CRP between elderly with depression or Alzheimer’s disease and controls without any psychiatric disorder. Convergent evidence of higher peripheral IL-1β, IL-6, TNF-α and CRP levels would support the psychoneuroimmunology theory as contributory to depressive syndromes and Alzheimer’s disease in elderly. Therefore, we conducted a systematic review, meta-analysis and meta-regression. Our hypothesis was that elderly with depression or Alzheimer’s disease had higher levels of IL-1β, IL-6, TNF-α and CRP than controls without psychiatric disorders. We aimed to identify factors that could influence the peripheral levels of these inflammatory markers.

## Methods

We followed the Preferred Reporting Items for Systematic Reviews and Meta- Analyses (PRISMA) guideline for conducting and reporting meta-analysis and systematic reviews^42^.

### Search Strategy

A comprehensive literature search was conducted through the major databases of PubMed, PsycINFO and Embase from inception to March 16, 2017. Searches including the following broad terms: cytokines, interleukins (interleukin, IL*, IL-1β, IL-6), tumor necrosis factor (TNF, tumor necrosis factor), c-reactive protein (CRP, c-reactive protein), elderly (late onset, late life, geriatric, elderly), mood disorders (depress*, depression, depressive, depressed, major depressive disorder, bipolar, manic-depressive) and Alzheimer’s disease (Alzheimer’s disease, Alzheimer, dementia, Alzheimer’s dementia, AD) where * indicates truncation^43^. A total of 2478 titles and abstracts were shortlisted, and references of the retrieved trials and review articles were also searched.

### Data extraction

The following information was extracted from each article: family name of the first author, country, year of publication, sample size (number of controls and number of elderly with depression or dementia), levels of pro-inflammatory cytokines or CRP, measure of depression or dementia, mean age, proportion of female gender, duration of education, severity of depression or dementia, proportion of diabetes, cerebrovascular disorder and cardiovascular disease. All information was recorded on a standardized data collection form and was checked by the first and last author. The quality of the 18 studies was appraised using adaptations of the Newcastle–Ottawa cohort scale for cross-sectional and case–control studies, which assessed the selection of study participants, comparability of outcome groups and assessment of outcome measures.

### Selection criteria

The two authors (AN and RCH) independently reviewed all abstracts and articles to evaluate whether the study would meet criteria for inclusion in the meta-analysis. If there was any disagreement between the authors about the inclusion of a study, a third author (MWZ) reviewed the study and made a decision. Studies were included if they fulfilled the following criteria: (1) Compared the blood levels of IL-1β, IL-6, TNF-α and CRP between elderly patients with depression or Alzheimer’s disease versus controls; (2) Patients with depression, Alzheimer’s disease and controls were at least 60 years old; (3) Study had sufficient information to calculate effect size. Studies were excluded if they: (1) studied patients with depression or Alzheimer’s disease who were younger than 60 years; (2) were animal studies; (3) did not measure levels of IL-1β, IL-6, TNF-α and CRP in the blood; (4) did not include a control group; (5) had a sample size of less than 30. If two studies were conducted in the same city within 5 years of publication, we would remove one of the studies that had smaller sample size to avoid overlapping of participants^44–46^. A total of 2444 articles were rejected because they did not meet the inclusion criteria. Thirty-four relevant articles were extracted and included for this meta-analysis.

### Statistical analyses

All statistical analysis was performed using the “metafor” function in R based on DerSimonian-Laird random-effects model^47^ and based on methods established by previous study comparing levels of biological markers between a disease group and controls without the disease^48^. The random-effects model was used because it assumes varying effect sizes between studies, because of differing study design and study population^49^. Differences in mean levels of IL-1β, IL-6, TNF-α and CRP between elderly who had Alzheimer’s disease or depression and controls were calculated using a standardized mean difference based on random-effect model. Random-effect model attempted to generalize findings beyond the included studies by assuming that the selected studies are random samples from a larger population^50^. Test of heterogeneity were conducted with the Q statistic that is distributed as a X^2^ variate under the assumption of homogeneity of effect sizes^43^. Between- study heterogeneity was assessed with the I^2^ statistic. As a guide, I^2^ values of 25% may be considered low, 50% as moderate, and 75% high^3,6^. Regression test was performed to examine publication bias if there are 10 or more studies for the outcome. Bonferroni adjustment for multiple testing in meta-analysis produced a rejection p-value of 0.05 divided by the total number of outcomes^51^. In this meta-analysis, there were 4 outcomes (IL-1β, IL-6, TNF-α and CRP). As a result, the rejection p value was 5/4 = 0.0125.

For results with considerable heterogeneity, meta-regression was performed to identify demographic and disease-related variables which might contribute to the heterogeneity if there were 5 or more studies with the variables. The regression coefficients and the associated *z* values and p values were reported in the meta-regression analysis^48^. Patient-related factors included mean age, gender, years of education, severity of depression or Alzheimer’s disease, history of diabetes, hyperlipidemia, coronary heart disease^52^.

## Results

**Figure 1:**
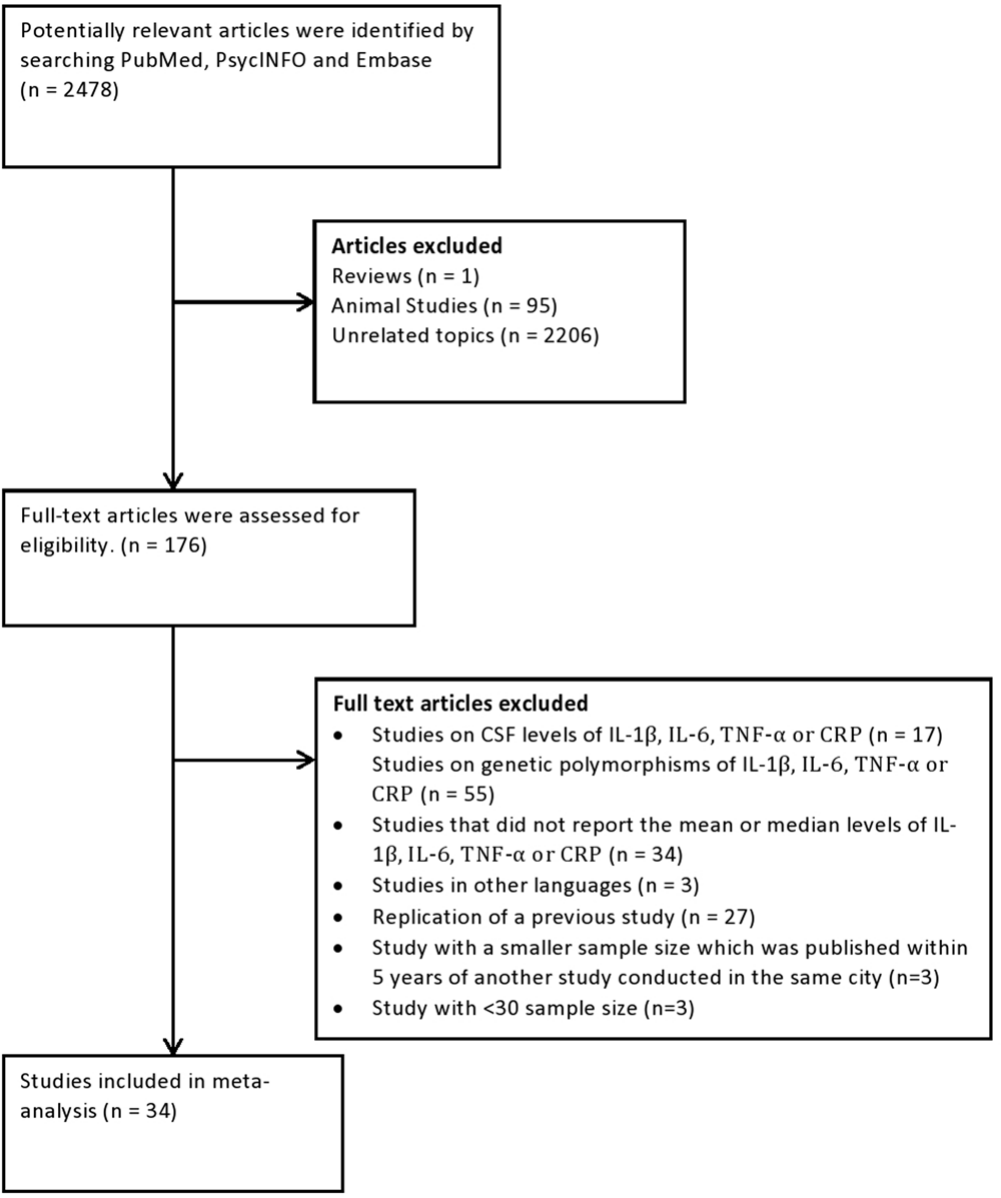
Flowchart of studies selected for review and inclusion for meta-analysis.

From an initial 2,478 potentially relevant articles, we assessed 176 full-text articles for eligibility. Finally, we included 34 articles in our analysis (Fig. 1). These studies comprising 2609 elderly with depression, 1645 elderly with Alzheimer’s disease and 14363 controls.

Majority of the studies were from western countries and characteristics of the population studied are shown in Table 1 and Table 2. Of these, five articles compared levels of IL-1β between depression and controls;^53–57^ seven articles compared IL-1β for Alzheimer’s disease and controls;^41,44,57–61^ nine articles compared IL-6 levels between depression and controls;^53,55,57,62–67^ eight articles compared IL-6 levels between Alzheimer’s disease and controls;^57,59,60,68–72^ five articles compared levels of TNF- α between depression and controls;^53,55,64,66,73^ ten articles compared the levels of TNF- α between Alzheimer’s disease and controls;^41,59,60,69–72,74–76^ nine articles compared the levels of CRP between depression and controls;^55,56,63–67,77,78^ eleven articles compared levels of CRP between Alzheimer’s disease and controls^60,69,79–87^. All the cytokine levels were converted to pg/ml.

**Table 1:** Characteristics of the Studies Comparing Peripheral Cytokine Levels Between Elderly with and without Depression Included in the Meta-analysis.

Author, year	Country	Peripheral pro-Inflammatory markers	Criteria for Depression	Sample size (N) (Depression/Controls)	Proportion of female gender (%) (Dementia/Controls)	Mean age (years) (Dementia/Controls)	Newcastle-Ottawa Scale Score (Maximum score = 9)
Forti, 2010 ^64^	Italy	IL-6, TNF-a, CRP	DSM-IV and ≥10 on GDS	93/870	74.2/52.9	81.4/ 73.8	7
Diniz, 2010 ^54^	Brazil	IL-1b	MDD on SCID	23/44	82.6/86.4	70.2 /69.5	5
Thomas, 2005^56^	UK	IL-1b, CRP	DSM-IV and ≥20 on Montgomery-Asberg DRS	19/21	68.4/57.1	76.4 /74.9	6
Torres, 2014^57^	Brazil	IL-1b, IL-6	DSM-IV, GDS, MINI-plus	13/18	61.5/50.0	74/ 83	5
Dimopoulos, 2007^62^	Greece	IL-6	DSM-IV and ≥10 on GDS	33/33	60.6 /60.6	65.8 / 65.4	7
Milaneschi, 2008^55^	Italy	IL-1b, IL-6, TNF-a, CRP	≥20 CES-D	213/778	75.1/ 50.6	77.5 / 74.3	7
Elderkin-Thompson, 2012^63^	US	IL-6, CRP	MDD on SCID and 15/17 on Hamilton DRS	42/45	66.7/75.6	69.7 / 69.2	5
Vogelzangs, 2014^67^	Netherlands	IL-6, CRP	DSM-IV and CIDI	358/131	66.2 / 61.1	70.6/ 70.1	6
Charlton, 2018^53^	US	IL-1b, IL-6, TNF-a	MDD on SCID and 15/17 on Hamilton DRS	24/34	66.7/ 61.7	67.2/ 70.2	6
Pennix, 2003^66^	US	IL-6, TNF-a, CRP	≥16 CES-D	145/2879	61.4 /51.0	74.0 /73.6	6
Nadrowski, 2016^65^	Poland	IL-6, CRP	>6 on GDS	1095/2423	Data not provided	Data not provided	7
Marinho, 2012^73^	Brazil	TNF-a	≥6 on GDS	3/50	Data not provided	Data not provided	6
Bremmer, 2008^77^	Netherlands	CRP	≥16 CES-D	38/1094	47.3/73.7	75.2/75.9	6
Kop, 2002^78^	United States	CRP	>10 CES-D	510/3750	Data not provided	Data not provided	6

**Table 2:** Characteristics of the Studies Comparing Peripheral Cytokine Levels Between Elderly with and without Dementia Included in the Meta-analysis.

Author, year	Study Country	Peripheral pro-Inflammatory markers	Criteria for dementia	Sample size (N) (Dementia/Controls)	Proportion of female gender (%) (Dementia/Controls)	Mean age (years) (Dementia/Controls)	Newcastle-Ottawa Scale Score (Maximum score = 9)
De Luigi, 2001^58^	Switzerland	IL-1b	NINCDS-ADRDA	12/43	Data not provided	Data not provided	5
Zuliani, 2007^59^	Italy	IL-1b, IL-6, TNF-a	NINCDS-ADRDA	60/42	65.0 /46.0	78.5/ 72.5	6
Yasutake, 2006^41^	Japan	IL-1b, TNF-a	NINCDS-ADRDA	60/33	66.7/ 75.8	77.9/ 71.1	5
Kalman, 1997^68^	UK	IL-6	NINCDS-ADRDA	26/24	65.4 / 75.0	75.4 /72.9	5
Villarreal, 2016^60^	Panama	IL-1b, IL-6, TNF-a, CRP	NINCDS-ADRDA	28/77	78.6/ 64.9	81.9/ 76.5	7
Torres, 2014^57^	Brazil	IL-1b, IL-6	NINCDS-ADRDA	35/18	71.4 / 50.0	81.0/ 83.0	5
Ciabattoni, 2007^69^	Italy	IL-6, TNF-a, CRP	NINCDS-ADRDA	44/44	56.8 / 61.4	73.0/ 75.0	5
Uslu, 2012^70^	Turkey	IL-6, TNF-a	NINCDS-ADRDA	28/23	64.3 / 56.5	68.4/ 66.2	5
Huang, 2013^71^	Taiwan	IL-6, TNF-a	DSM-IV	28/19	14.3 / 26.3	83.0 / 79.9	5
Alvarez, 2007^74^	Spain	TNF-a	NINCDS-ADRDA	141/30	70.2 / 66.7	75.3 / 68.9	5
Bonotis, 2008^72^	Greece	IL-6, TNF-a	NINCDS-ADRDA	19/21	57.9 / 52.4	75.1/ 71.2	5
Mulder, 2010 ^79^	Netherlands	CRP	NINCDS-ADRDA	140/30	55.0 / 57.0	66.0/ 65.0	7
O’Bryant, 2010 ^80^	US	CRP	NINCDS-ADRDA	192/174	50.0 / 63.2	75.8/ 70.7	6
Lawlor, 1996^81^	Ireland	CRP	NINCDS-ADRDA	17/15	70.6/ 80.0	74.9/ 68.5	5
Yarchoan, 2013^87^	US	CRP	NINCDS-ADRDA	203/117	58.0 / 63.0	74.5/ 69.9	8
Lepara, 2009^82^	Yugoslavia	CRP	NINCDS-ADRDA	30/30	80.0 / 73.3	80.0 /77.5	5
Mancinella, 2009^83^	Italy	CRP	NINCDS-ADRDA	34 99	58.0 / 65.0	83.4/ 83.0	5
Davis, 2009^84^	Trinidad and Tobago	CRP	NINCDS-ADRDA	18/51	Data not provided	Data not provided	5
Solerte, 2000^75^	Italy	TNF-a	NINCDS-ADRDA	22/15	Data not provided	76.5 /77.0	5
Hsu, 2017^85^	Taiwan	CRP	ICD-9	260 /1176	51.9 /48.0	74.1/ 73.0	7
Licastro, 2000^86^	Italy	CRP	NINCDS-ADRDA	145/51	62.8 /60.8	75.0/ 78.0	5
Laske, 2011^76^	Germany	TNF-a	NINCDS-ADRDA	45/30	Data not provided	Data not provided	5
Forlenza, 2009^61^	Brazil	IL-1b	NINCDS-ADRDA	58/31	82.8/ 64.5	76.3/ 69.9	5

### Meta-Analysis for Elderly with and without Depression

**Figure 2:**
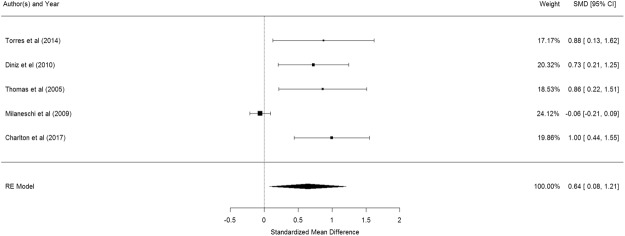
Forest plot of studies comparing peripheral IL-1β levels between elderly suffering from depression and controls.

Figure 2 shows the results of the 5 studies that compared the peripheral IL-1β levels between elderly suffering from depression and controls^53–57^. Elderly with depression were significantly higher in their peripheral IL-1β levels than control participants (pooled SMD with random-effects model: 0.642, 95% CI: 0.078–1.206, z = 2.232, p = 0.026). A significant level of between-study heterogeneity was found (τ^2^ = 0.337, Q = 29.16, df = 4, p < 0.001, I^2^ = 86.28%). When we undertook meta-regression to explore the impact of a priori sources of heterogeneity, no significant association was observed for age (β = −0.023 p = 0.767) and proportion of women (β = 1.070, p = 0.677).

**Figure 3:**
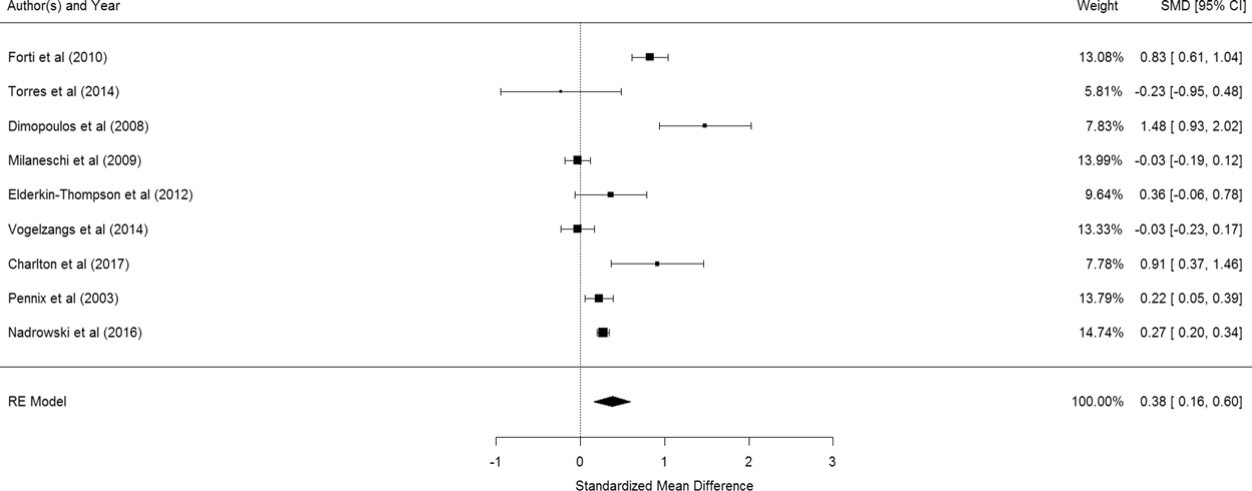
Forest plot of studies comparing the peripheral IL-6 levels between elderly suffering from depression and controls.

Figure 3 shows the results of the 9 studies that compared the peripheral IL-6 levels between elderly suffering from depression and controls^53,55,57,62–67^. Elderly with depression were significantly higher in their peripheral IL-6 levels than control participants (pooled SMD with random-effects model: 0.377, 95% CI: 0.156–0.598, z = 3.348, p < 0.001). A significant level of between-study heterogeneity was found (τ^2^ = 0.085, Q = 75.75, df = 8, p < 0.001, I^2^ = 89.44%). Meta-regression shows that mean age of participants is a significant moderator (β = −0.093, p = 0.0363) but proportion of women is not (β = 1.108, p = 0.560). After adjustment of age, the adjusted pooled SMD became 0.2895. Publication bias was not detected (p = 0.1909).

**Figure 4:**
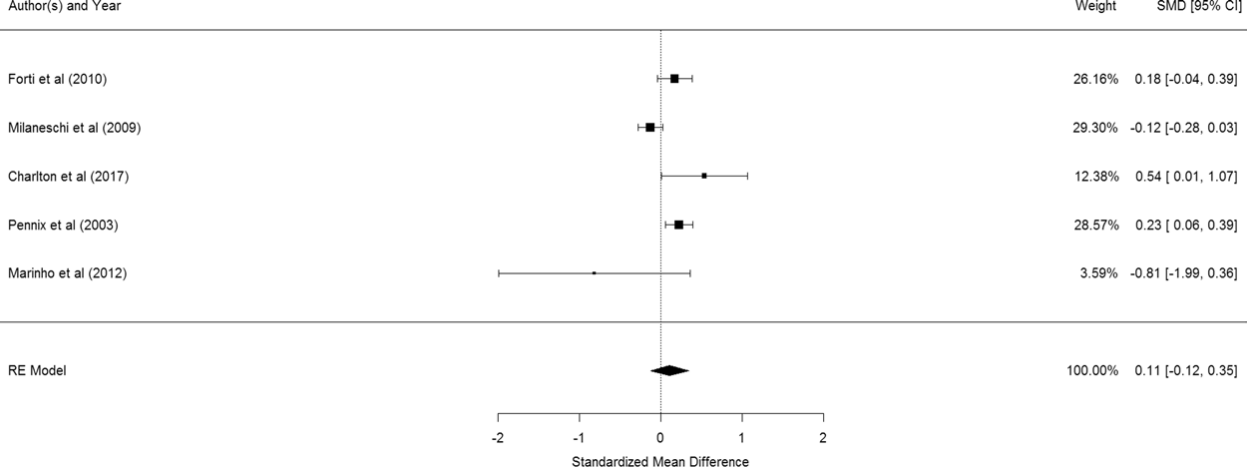
Forest plot of studies comparing peripheral TNF- α levels between elderly suffering from depression and controls.

Figure 4 shows the results of the 5 studies that compared the peripheral TNF- α levels between elderly suffering from depression and controls^53,55,64,66,73^. There was no significant difference in peripheral TNF- α levels between elderly with and without depression (pooled SMD with random-effects model: 0.112, 95% CI: −0.123–0.348, z = 0.968, p = 0.351). A significant level of between-study heterogeneity was found (τ^2^ = 0.044, Q = 15.80, df = 4, p = 0.003, I^2^ = 74.68%). Meta-regression was not conducted as there were too few studies.

**Figure 5:**
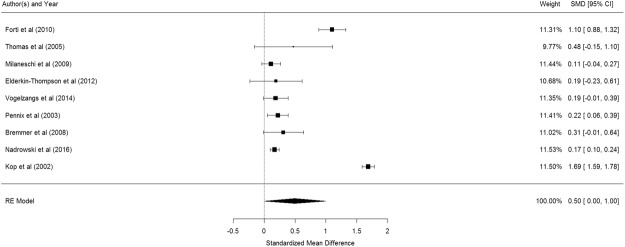
Forest plot of studies comparing peripheral CRP levels between elderly suffering from depression and controls.

Figure 5 shows the results of the 9 studies that compared the peripheral CRP levels between elderly suffering from depression and controls^55,56,63–67,77,78^. Elderly with depression were nominal higher in their peripheral CRP levels than control participants but not statistically significant (pooled SMD: 0.499, 95% CI: 0.001–0.999, z = 1.961, p = 0.050). A significant level of between-study heterogeneity was found (τ^2^ = 0.562, Q = 698.5, df = 8, p < 0.001, I^2^ = 98.9%). No significant association was revealed for mean age of participants (β = 0.049, p = 0.499) or proportion of elderly women (β = −0.012, p = 0.650) from meta-regression. Publication bias was not detected (p = 0.734).

Bonferroni adjustment for multiple testing produced a rejection p-value of 0.0125. This would mean that the outcome involving peripheral levels of IL-1β was no longer statistically significant. However, the outcome involving peripheral levels of IL-6 remained statistically significant.

### Meta-Analysis for Elderly with and without Alzheimer’s disease

**Figure 6:**
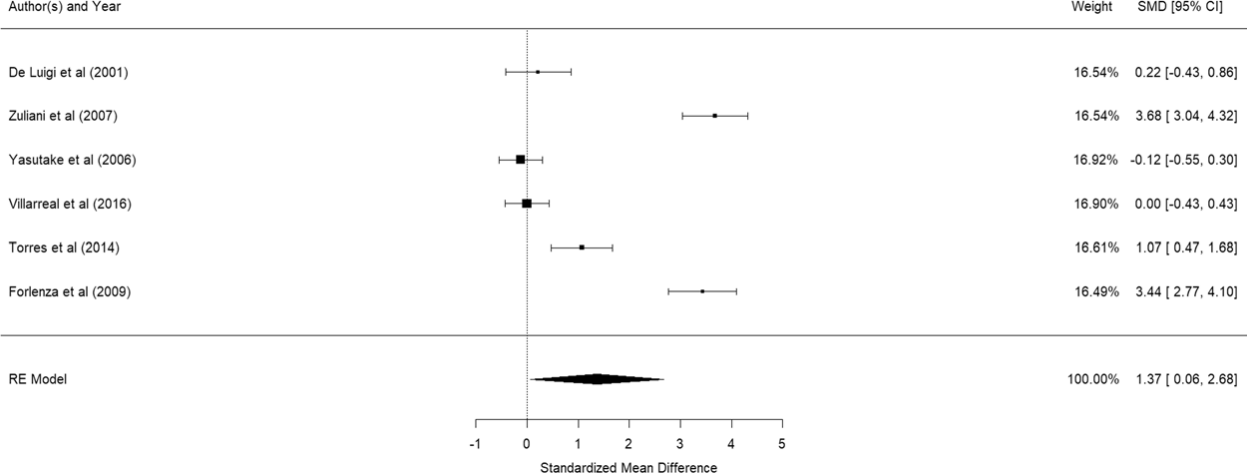
Forest plot of studies comparing peripheral IL-1β levels between elderly suffering from Alzheimer’s disease and controls.

Figure 6 shows the results of the 6 studies that compared the peripheral IL-1β levels between elderly suffering from Alzheimer’s disease and controls^41,57–61^. Elderly with Alzheimer’s disease were significantly higher in their peripheral IL-1β levels than control participants (pooled SMD: 1.37, 95% CI: 0.06–2.68, z = 1.98, p = 0.041). A significant level of between-study heterogeneity was found (τ^2^ = 2.31, Q = 175.54, df = 7, p < 0.001, I^2^ = 96.01%). Meta-regression showed that mean age (β = −0.72, p = 0.247) and proportion of elderly women (β = 0.031, p = 0.626) were not statistical significant. Regression for MMSE was not done as there were less than 5 studies with MMSE score.

**Figure 7:**
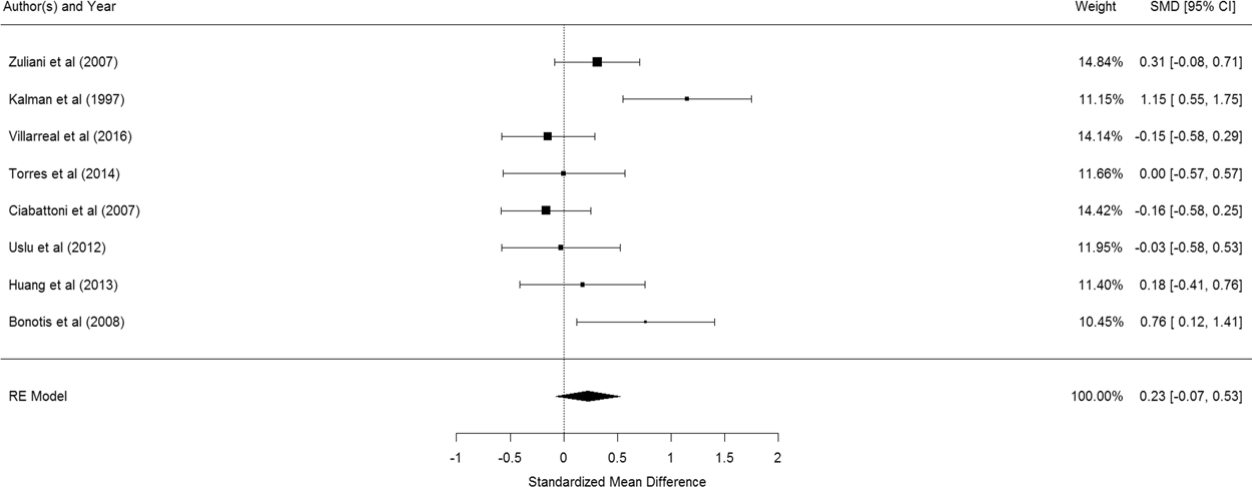
Forest plot of studies comparing peripheral IL-6 levels between elderly suffering from Alzheimer’s disease and controls.

Figure 7 shows the results of the 8 studies that compared the peripheral IL-6 levels between elderly suffering from Alzheimer’s disease and controls^57,59,60,68–72^. There was no significant difference in peripheral IL-6 levels between elderly with and without Alzheimer’s disease (pooled SMD with random-effects model: 0.228, 95% CI: −0.074–0.528, z = 1.482, p = 0.138). A significant level of between-study heterogeneity was found (τ^2^ = 0.117, Q = 19.36, df = 7, p = 0.007, I^2^ = 63.84%). Meta-regression showed that MMSE score was statistically significant (β = −0.1048, p = 0.010) while no significant association was observed for mean age (β = −0.098, p = 0.819) and proportion of women (β = 0.002, p = 0.853).

**Figure 8:**
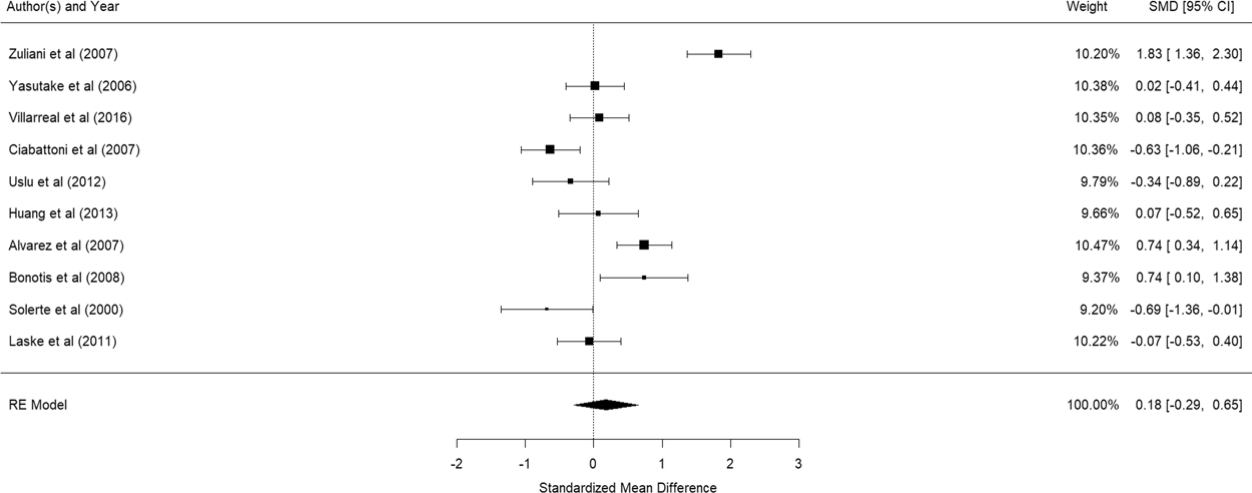
Forest plot of studies comparing peripheral TNF-α levels between elderly suffering from Alzheimer’s disease and controls.

Figure 8 shows the results of the 10 studies that compared the peripheral TNF-α levels between elderly suffering from Alzheimer’s disease and controls^41,59,60,69–72,74–76^. There was no significant difference in peripheral TNF-α levels between elderly with and without Alzheimer’s disease (pooled SMD with random-effects model: 0.18, 95% CI: -0.29–0.65, z = 0.338, p = 0.451). A significant level of between-study heterogeneity was found (τ^2^ = 0.542, Q = 95.14, df = 11 p < 0.001, I^2^ = 88.44%). Meta-regression revealed that mean age (β = 0.0466, p = 0.654), proportion of women (β = −0.031, p = 0.594), and MMSE (β = −0.2466, p = 0.2908) were not statistically significant. No publication bias was detected (p = 0.0802).

**Figure 9:**
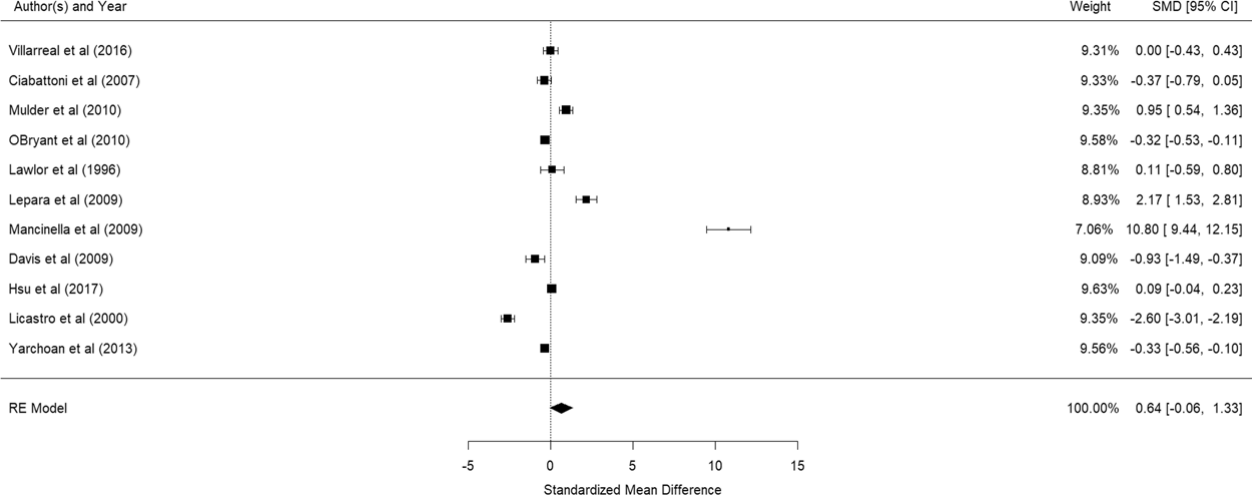
Forest plot of studies comparing peripheral CRP levels between elderly suffering from Alzheimer’s disease and controls.

**Figure 10:**
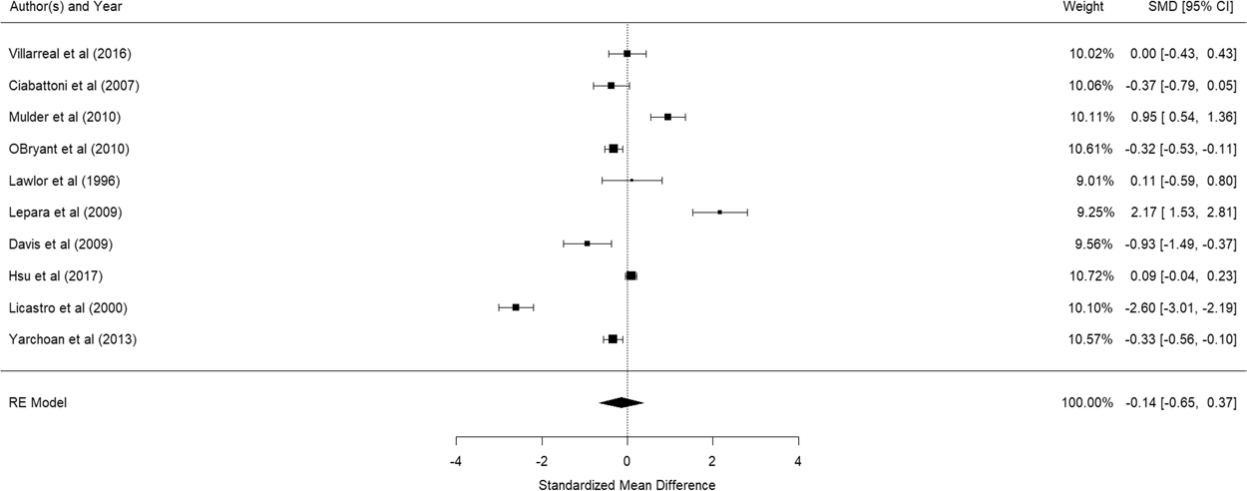
Forest plot of studies comparing peripheral CRP levels between elderly suffering from Alzheimer’s disease and controls (after exclusion of a study with extremely large effect size)

Figure 9 shows the results of the 11 studies that compared the peripheral CRP levels between elderly suffering from Alzheimer’s disease and controls^60,69,79–87^. There was no significant difference in peripheral CRP levels between elderly with and without Alzheimer’s disease (pooled SMD with random-effects model: 0.638, 95% CI: −0.055–1.331, z = 1.804, p = 0.0712). A significant level of between-study heterogeneity was found (τ^2^ = 1.293, Q = 492.4, df = 10, p < 0.001, I^2^ = 97.97). Meta-regression revealed that mean age (β = 0.0796, p = 0.527) and proportion of women (β = −0.035, p = 0.274), were not statistically significant. Publication bias was detected (p < 0.001). As Mancinella’s study reported an extremely large effect, a sensitivity analysis was conducted by excluding it in the analysis. Without this study, the pooled SMD became −0.142 (95% CI: −0.654–0.370) (See Fig. 10) and publication bias was no longer detected (p = 0.368).

Bonferroni adjustment for multiple testing produced a rejection p-value of 0.0125. This would mean that the outcome involving peripheral levels of IL-1β was no longer statistically significant.

## Discussion

In this meta-analysis, we found that elderly with depression had significantly higher peripheral levels of IL-1β (p = 0.026), IL-6 (p < 0.001) but not TNF-α (p = 0.351) and CRP (p = 0.05). After Bonferroni adjustment, only peripheral levels of IL-1β remained significantly higher in elderly with depression than controls. However, elderly with Alzheimer’s disease only had significantly higher peripheral levels of IL-1β (p = 0.047) but not IL-6 (p = 0.138), TNF-α (p = 0.735) or CRP (p = 0.0712). After Bonferroni adjustment, none of the above inflammatory markers remained significant. Heterogeneity was noted in the studies for depression and Alzheimer’s disease. Factors such as age range of the elderly, proportion of gender in subjects, severity of the condition (depression or Alzheimer’s disease), presence and extent of medical comorbidities, study design and methodical variance which includes the types of assay kits used to measure cytokines, may contribute to the observed heterogeneity. Nevertheless, it is not feasible to match for all these factors in the meta-analysis. In this meta-analysis, there was no publication bias. Our results with Bonferroni adjustment are different from a previous meta-analysis which was based on 7 studies and found that higher levels of IL-6 and CRP were associated with increased risk of all-cause dementia^38^. Furthermore, this previous meta-analysis did not study IL-1β and TNF-α^38^. Ravaglia *et al*.^88^ reported that peripheral inflammatory markers are associated with increased risk of vascular dementia but not Alzheimer’s disease. This postulation might explain why this meta-analysis found that all peripheral inflammatory markers were not increased in Alzheimer’s disease. Many previous studies examining blood cytokine levels and cognitive impairment were also inconclusive in results^89^ and this could possibly be attributed by lack of longitudinal data on cytokine expression, inadequate methodological standardization and standardization of patient characteristics. Further prospective studies are required to study the role of each proinflammatory cytokine in different types of dementia and correlate with clinical features, neuroimaging and presence of beta amyloid and neurofibrillary tangles. Our results suggest that peripheral pro-inflammatory marker IL-6 is more likely to be increased in depression as compared to Alzheimer’s disease in elderly. The increased peripheral IL-6 levels in elderly with depression indicate vascular risk factors and atherosclerosis^59^ which can contribute to depression. Primary prevention strategies targeting metabolically mediated comorbidity (e.g., cardiovascular disease) may prevent depression in elderly^90^. Previous study revealed the potential therapeutic effects of antidepressant fluoxetine by reducing central and peripheral levels of IL-6 in the alleviation of depressive symptoms^91^.

In this meta-analysis, elderly with depression or Alzheimer’s disease were found to have significantly higher peripheral IL-1β levels before Bonferroni adjustment. IL-1β is produced by macrophages, endothelial cells, and astrocytes^59^. IL-1β was found to be a significant risk factor for the development of depressive symptoms^92^. IL-1β crosses the blood brain barrier^93^ and subsequently alters the HPA-axis^11^. IL-1β reduces the re-uptake of serotonin from the synaptic cleft resulting in depressive symptoms^94^. For Alzheimer’s disease, Griffin *et al*.^95^ found that IL-1β plays an important role in causing neurodegenerative changes. IL-1β drives the synthesis of β-amyloid precursor protein, resulting in the production and deposition of β-amyloid plaques in the brain of patients with Alzheimer’s disease^95,96^. It is also involved in tau phosphorylation^97^, a key pathogenic process in Alzheimer’s disease^7^.

IL-6 is produced by macrophages, T-lymphocytes, endothelial cells, smooth muscle cells and adipocytes^59^. IL-6 modulates the HPA-axis which stimulates CRH, ACTH and cortisol^98^. IL-1β and IL-6 could also work together and synergistically induce systemic immune response, leading to psycho-neuro-immunological changes in patients suffering from depression^98^. IL-6 also induces production of indoleamine 2,3-dioxygenase (IDO), leading to a decrease in tryptophan and the production of tryptophan catabolites (TRYCATs), and this is associated with depressive symptoms^99^. In contrast, peripheral IL-6 level was not significantly higher in elderly with Alzheimer’s disease as compared to controls. This finding could be supported by previous research which found that elevations observed in peripheral IL-6 levels preceded the onset of Alzheimer’s disease but not during the course of Alzheimer’s disease^70^.

CRP is an acute-phase protein of hepatic origin that increases following IL-6 secretion by macrophages and T cells. In this meta-analysis, there was no significant difference in peripheral CRP levels in elderly with depression and Alzheimer’s disease as compared to controls before and after Bonferroni’s correction. Eriksson *et al*.^100^ proposed that increased peripheral levels of CRP in mid-life played a more detrimental role leading to Alzheimer’s disease but not in older people. Our findings revealed that peripheral CRP level was not increased but IL-1β was significantly raised in elderly with Alzheimer’s disease as compared to controls before Bonferroni adjustment. Licastro *et al*.^86^ postulated that the IL-1β increment in elderly with Alzheimer’s disease might be due to central immune response.

TNF-α is produced by macrophages, adipocytes and astrocytes^70^. In this meta-analysis, peripheral levels of TNF-α were not significantly increased in elderly with depression as well as Alzheimer’s disease as compared to controls before and after Bonferroni adjustment. Firstly, our findings might suggest that peripheral TNF-α is a less sensitive marker as compared to IL-1β and IL-6. Secondly, our findings support previous postulation that the levels of peripheral TNF- α was found to be significantly lower in mild to moderate Alzheimer’s disease as compared to severe Alzheimer’s disease^101^. When we included participants with different severity of Alzheimer’s disease, the peripheral TNF – α levels were not significantly higher than controls.

### Limitations

This meta-analysis has several limitations. Firstly, one major limitation is lack of repeated measurements for inflammatory markers in elderly with depression and Alzheimer’s disease in this meta-analysis. We cannot ignore the limitations of performing a meta-analysis on cross-sectional studies. The causality of high levels of peripheral inflammatory markers in elderly with depression cannot be established^102^. Based on the current study, it is not possible to delineate whether inflammation plays a causal role in depression or dementia, or if inflammatory processes in cardiovascular diseases that are common in the elderly lead to increased depressive symptoms in elderly or vice versa. Secondly, this meta-analysis focused on peripheral inflammatory markers which might not reflect inflammatory process in the central nervous system.

## Conclusion

In conclusion, our meta-analysis found that elderly with depression had elevated peripheral levels of IL-1β, IL-6 but not CRP and TNF-α, and elderly with Alzheimer’s disease only had increased peripheral levels of blood IL-1β. After correction for multiple testing, only peripheral levels of IL-6 remained significantly higher in elderly with depression. The involvement of peripheral IL-6 in elderly with depression reflects a high proportion of metabolic comorbidities which are modifiable. Further studies are necessary to evaluate the association between high peripheral IL-6 levels and the subsequent risk of developing depression in old age.
